# Relationship between Insulin Resistance and Circulating Stem and Progenitor Cells in Newly-diagnosed Youth-onset Type 2 Diabetes

**DOI:** 10.2174/0118715303447742260214154721

**Published:** 2026-04-08

**Authors:** Shiqi Sun, Zhiheng Sun, Xiaoxue Wu, Qi Huang, Jiazhong Sun

**Affiliations:** 1 Department of Laboratory Medicine, The Sixth Hospital of Wuhan, Affiliated Hospital of JiangHan University, Wuhan 430072, China;; 2 School of Health and Nursing, Wuchang University of Technology, Wuhan 430071, China;; 3 Department of Endocrinology, Zhongnan Hospital of Wuhan University, Wuhan 430071, China

**Keywords:** Insulin resistance, circulating stem cells, endothelial progenitor cells, youth-onset type 2 diabetes, vascular endothelial dysfunction, hemoglobin

## Abstract

**Introduction:**

To investigate the relationship between insulin resistance (IR) and circulating stem cells and endothelial progenitor cells (EPCs) in youth-onset type 2 diabetes mellitus (T2DM).

**Methods:**

A total of 392 newly diagnosed T2DM patients aged 12–25 years were enrolled. General data, medical history, and anthropometric indices were collected. Blood glucose, lipid profiles, and glycated hemoglobin (HbA1c) were measured. IR was assessed using the homeostatic model assessment of insulin resistance (HOMA-IR). Circulating stem cells (CD34^+^ cells) and EPCs (CD34^+^ kinase insert domain receptor [KDR]+ cells) were quantified *via* flow cytometry.

**Results:**

When stratified by HOMA-IR tertiles, significant differences were observed across groups in CD34^+^ stem cells, CD34^+^ KDR^+^ EPCs, body mass index (BMI), systolic blood pressure (SBP), diastolic blood pressure (DBP), triglycerides (TG), total cholesterol (TC), low-density lipoprotein cholesterol (LDL-C), and high-density lipoprotein cholesterol (HDL-C) (all *P* < 0.05 or *P* < 0.01). No significant differences were detected in age, sex, fasting plasma glucose (FPG), or HbA1c. Correlation analysis demonstrated inverse associations of CD34^+^ stem cells (r = −0.453) and CD34^+^ KDR^+^ EPCs (r = −0.531) with HOMA-IR (both *P* < 0.01), while positive correlations were observed with BMI (-0.327; -0.342), SBP (-0.261; -0.246), DBP (-0.213; -0.269), TG (-0.182; -0.199), TC (-0.221; -0.258), and LDL-C (-0.234; -0.293). HDL-C showed a positive correlation (r = 0.137, r = 0.247, both *P* < 0.05). No significant associations were found with age, sex, FPG, or HbA1c. In multivariate regression analysis, HOMA-IR remained independently associated with reduced CD34^+^ stem cells (adjusted β = 1.846, 95% CI: 0.723–4.209, *P* < 0.05) and CD34^+^ KDR^+^ EPCs (adjusted β = 1.985, 95% CI: 0.856–4.843, *P* < 0.05) after adjusting for BMI, blood pressure, and lipid parameters.

**Discussion:**

Our results indicate that insulin resistance plays a crucial, reversible role in the development of early vascular damage among adolescents with type 2 diabetes. This highlights the importance of implementing early interventions aimed at improving insulin sensitivity in this vulnerable population.

**Conclusion:**

HOMA-IR was independently associated with reduced levels of circulating stem cells and EPCs in youth-onset type 2 diabetes, indicating its role as an independent risk factor for vascular endothelial dysfunction in this population.

## 1 INTRODUCTION

Cardiovascular complications are the most common and deadly comorbidities in people with type 2 diabetes mellitus (T2DM), responsible for more than 50% of diabetes-related deaths worldwide [1]. It is crucial to highlight that decreases in the levels of circulating stem cells and endothelial progenitor cells (EPCs) are predictive of cardiovascular outcomes across various populations [2]. Notably, CD34^+^ cells are indicative of the future onset of microangiopathy and macroangiopathy in diabetic patients [3, 4]. These findings highlight the importance of using cellular biomarkers for risk stratification in cardiovascular diseases (CVD).

The increasing prevalence of pediatric T2DM stands in sharp contrast to the limited understanding of its cardiovascular trajectory. Recent studies indicate that adolescents with T2DM experience vascular endothelial dysfunction and smooth muscle hyperplasia accompanied by collagen deposition at an early stage of the disease. These changes contribute to the accelerated formation of atherosclerotic lesions [5, 6]. Clinical imaging has revealed the swift advancement of subclinical atherosclerosis in young individuals withT2DM. Specifically, these youths demonstrate a 23% increase in carotid intima-media thickness (CIMT) compared to age-matched controls, and the presence of detectable coronary artery calcification in 18% of cases within five years following their diagnosis [7, 8]. These findings underscore a unique and aggressive vasculopathy phenotype that demands immediate investigation.

Although insulin resistance (IR) is a key factor in the diagnosis of T2DM and pediatric metabolic dysfunction, its role as an independent contributor to cardiovascular disease (CVD) risk is still debated. In non-diabetic populations, obesity-driven IR correlates strongly with arterial stiffness and preclinical atherosclerosis [9]. Paradoxically, in established T2DM, hyperglycemia, dyslipidemia, and hypertension may overshadow IR's direct vascular effects. Therefore, to address this knowledge gap, this study focused on newly diagnosed T2DM patients aged 12–25 years to investigate the relationship between IR and circulating stem cells and EPCs, aiming to provide insights for CVD prevention and management in youth-onset type 2 diabetes.

Therefore, the primary objective of this cross-sectional study was to investigate the independent relationship between insulin resistance (assessed by HOMA-IR) and the levels of circulating stem cells (CD34^+^ cells) and endothelial progenitor cells (CD34^+^KDR^+^ EPCs) in newly diagnosed youth-onset type 2 diabetes.

## 2 MATERIALS AND METHODS

### 2.1 Study Subjects

A total of 392 newly diagnosed youth-onset type 2 diabetes patients were enrolled from the Department of Endocrinology or Pediatrics at Wuhan University Zhongnan Hospital between January 2020 and December 2023, be naïve to antihyperglycaemic drugs, and had not previously received any systematic diabetes related medical advice and interventions. The age range was 12–25 years (mean±SD: 19.5±4.6 years), with 208 males and 184 females. Diagnostic criteria: T2DM diagnosis followed the Chinese Guidelines for Prevention and Treatment of Type 2 Diabetes Mellitus (2020 Edition) and the American Diabetes Association criteria [10]. Exclusion criteria: Positive diabetes autoantibodies, specific types of diabetes, requirement for insulin therapy, acute diabetic complications, concurrent autoimmune thyroid diseases or other autoimmune disorders, and pregnancy. The study protocol was approved by the Ethics Committee of Wuhan University Zhongnan Hospital (Approval Number: 2023LSG-017). Written informed consent was obtained from patients aged ≥18 years, while parental/guardian consent was provided for those <18 years.

### 2.2 Sample Size Calculation

An initial power analysis was conducted using G*Power (version 3.1). According to the preliminary data, we hypothesized a moderate effect size (f = 0.25) for the relationship between HOMA-IR and CD34 ^+^ cell levels, setting the significance level at α = 0.05 and aiming for a statistical power of 0.80. The analysis suggested that a minimum of 352 participants would be necessary for the sample size. To account for a potential 10% dropout rate or incomplete data, we aimed to recruit a minimum of 390 participants. Our final enrollment of 392 participants successfully reached this target.

### 2.3 Anthropometric Indicators

 General demographic data and medical history were collected from all participants. Height and weight were measured in meters and kilograms, respectively. Body mass index (BMI) was calculated as weight (kg)/height (m)^2^. Supine blood pressure (mmHg) was measured following the Chinese Clinical Practice Guideline for Hypertension (2022 Edition) [11]. Three consecutive readings were taken, and the mean value was used for analysis.

### 2.4 Biochemical Parameter Testing

 Laboratory Assessments: Fasting Plasma Glucose (FPG); Fasting Insulin (FINS); Total Cholesterol (TC); Triglycerides (TG); High-Density Lipoprotein Cholesterol (HDL-C) ; Low-Density Lipoprotein Cholesterol (LDL-C); Glycated Hemoglobin (HbA1c). Glucose and Lipid Profiles: Measured *via* enzymatic assays; HbA1c: Quantified using ion-exchange high-performance liquid chromatography (HPLC); FINS: Detected *via* chemiluminescence immunoassay (CLIA). Homeostatic Model Assessment of Insulin Resistance (HOMA-IR): HOMA-IR= FINS (μIU/mL) ×FPG (mmol/L)/22.5.

### 2.5 Flow Cytometry

Circulating stem/progenitor cells were analyzed by flow cytometry as previously described [4, 12]. Peripheral blood mononuclear cells (PBMCs) were isolated *via* density gradient centrifugation and stained with fluorescently labeled monoclonal antibodies against CD34 (clone 581, BD Biosciences, San Jose, CA, USA) and KDR (clone 89106, R&D Systems, Minneapolis, MN, USA). CD34^+^ cells were gated within the PBMC population using forward/side scatter parameters, followed by quantification of CD34^+^KDR^+^ endothelial progenitor cells (EPCs). A minimum of 1×10^6^/ events per sample was acquired on a BD FACSCanto II flow cytometer (BD Biosciences) equipped with FACSDiva software (v8.0.1). Absolute cell counts (cells/μL blood) were calculated using a dual-platform method: cell frequencies per 1×10^6^/events were multiplied by total leukocyte counts (×10^3^/μL) measured by an automated hematology analyzer (Sysmex XN-1000) [13]. To ensure consistency, all analyses were performed by two trained operators using standardized protocols. Despite changes in KDR antibody lots during the study, pre-experiment validation confirmed comparable staining efficiency (<5% deviation). Reproducibility was evaluated by performing measurements in triplicate, resulting in intra-assay coefficients of variation of 6.1% for CD34^+^ cells and 11.9% for CD34^+^KDR+ endothelial progenitor cells (EPCs).

### 2.6 Statistical Analysis

This cross-sectional observational study employed SPSS 19.0, a statistical software package commonly used in social sciences, to conduct the data analysis. Participants were divided into three groups according to the tertiles of HOMA-IR: H1 (HOMA-IR < 2.8), H2 (HOMA-IR 2.8–4.3), and H3 (HOMA-IR > 4.3). Continuous variables following a normal distribution were expressed as mean ± standard deviation (xˉ±s), and comparisons between groups were conducted using Analysis of Variance (ANOVA). Categorical variables were expressed as proportions or percentages and analyzed using the Chi-squared test for comparison. The Pearson correlation coefficient was utilized to evaluate the relationships between the variables. Multivariable linear regression was employed to examine the association between HOMA-IR and levels of circulating stem cells (CD34^+^ cells) as well as endothelial progenitor cells (CD34^+^ KDR^+^ EPCs). The analysis was adjusted for various cardiovascular risk factors, including systolic and diastolic blood pressure, lipid profile components (triglycerides, total cholesterol, LDL-C, and HDL-C), and glucose concentrations. A two-tailed *P*-value < 0.05 was considered statistically significant.

## 3 RESULTS

### 3.1 Comparative Analysis of Circulating Stem Cells, Endothelial Progenitor Cells, and Cardiometabolic Risk Parameters Among HOMA-IR Tertile Groups

After stratifying the participants into HOMA-IR tertile groups (H1, H2, H3), statistically significant differences were observed across the groups in terms of CD34^+^ cells, CD34^+^ KDR^+^ EPCs, BMI, SBP, DBP, TG, TC, LDL-C, and HDL-C (all *P* < 0.05 or *P* < 0.01). In contrast, no significant intergroup differences were observed for age and sex (all *P* > 0.05). Notably, although FPG and HbA1c showed an increasing trend across the tertiles, these differences were not statistically significant (all *P* > 0.05), as outlined in Table **1**.

### 3.2 Analysis of the Correlation between HOMA-IR and Other Risk Factors with Circulating Stem Cells and EPCs

Circulating stem cells and EPCs showed consistent inverse correlations with a range of metabolic and cardiovascular risk factors. Specifically, both cell types exhibited significant negative associations with the HOMA-IR (circulating stem cells: r = -0.453; EPCs: r = -0.330), BMI (-0.327; -0.342), SBP (-0.261; -0.246), DBP (-0.213; -0.269), TG (-0.182; -0.199), TC (-0.221; -0.258), and LDL-C (-0.234; -0.293) (all *P* < 0.05 or *P* < 0.01). In contrast, both cell types exhibited a significant positive correlation with HDL-C (circulating stem cells: r = 0.137; EPCs: r = 0.247, both *P* < 0.05). No significant correlations were found with age, gender, FPG, or HbA1c (r range: 0.008–0.019, all *P* > 0.05).

After adjusting for BMI, blood pressure, and lipid profiles, the partial correlation between HOMA-IR and circulating stem cells was reduced but still maintained statistical significance (r = -0.173, *P* < 0.05). Interestingly, the adjusted association for EPCs grew stronger (r = -0.259, *P* < 0.05), suggesting a consistent and independent connection between insulin resistance and lower levels of progenitor cells, even when accounting for metabolic confounders.

### 3.3 Impact of Insulin Resistance and Cardiometabolic Risk Factors on Circulating Progenitor Cell Populations

Linear regression analyses, with circulating stem cells and EPCs as dependent variables, identified HOMA-IR as an independent predictor. In unadjusted models, HOMA-IR showed strong inverse associations with progenitor cell levels: β = -3.543 (95% CI: -5.989 to -2.001, *P* < 0.01) for circulating stem cells and β = -4.633 (95% CI: -6.011 to -1.981, *P* < 0.01) for EPCs. After adjusting for metabolic confounders, including BMI, systolic and diastolic blood pressure, triglycerides, total cholesterol, LDL-C, and HDL-C, the associations remained statistically significant but were somewhat reduced. Specifically, the β coefficient was 1.846 (95% CI: 0.723 to 4.209, *P* < 0.05) for circulating stem cells and 1.985 (95% CI: 0.856 to 4.843, *P* < 0.05) for EPCs.

The consistent negative association between HOMA-IR and progenitor cell counts, even after multivariable adjustment (*P* < 0.05 for both cell types), indicates that insulin resistance may independently contribute to diminished vascular regenerative capacity, irrespective of dyslipidemia and hypertension. These results are consistent with well-established pathophysiological mechanisms, in which metabolic dysfunction promotes cardiovascular risk through two key pathways: (1) oxidative stress-induced endothelial injury and (2) the inhibition of bone marrow-derived progenitor cell mobilization, collectively compromising vascular repair mechanisms.

## 4 DISCUSSION

Meta-analyses have shown that decreased levels of circulating stem cells and endothelial progenitor cells (EPCs) are strongly linked to the development of macrovascular complications in people with diabetes. These complications include coronary artery disease, peripheral arterial disease, and cerebrovascular events [2-4]. This correlation could arise from the crucial role that circulating stem cells and EPCs play in vascular repair and maintaining endothelial homeostasis. In individuals with diabetes, the processes of EPCs mobilization, differentiation, and function are compromised due to hyperglycemia, insulin resistance, and chronic inflammation. This impairment results in reduced vascular regeneration and a faster progression of atherosclerosis. In clinical settings, the depletion of EPCs has been suggested as a biomarker for predicting cardiovascular risk in individuals with diabetes. This highlights its potential value in strategies aimed at early intervention.

Endothelial dysfunction has become a key risk factor contributing to increased cardiovascular morbidity in populations affected by obesity-related insulin resistance and T2DM. This connection becomes increasingly urgent as the global prevalence of early-onset T2DM in children and adolescents continues to rise, driving more intensive research into cardiovascular risk profiles within this age group. Recent studies indicate that adolescents suffering from obesity or obesity-related T2DM exhibit a faster progression of arterial abnormalities [13]. These abnormalities are accompanied by detectable declines in endothelial function and noticeable structural changes in the carotid vasculature [14]. At the heart of this phenomenon, from a pathophysiological perspective, is insulin resistance, which serves as both a key trigger and a perpetuating factor in the progression of T2DM. Notably, clinical investigations establish insulin resistance as an independent predictor of atherosclerotic changes in adult populations. The impact on blood vessels extends to cellular mediators, where circulating stem cells and EPCs have shown prognostic significance for future microvascular and macrovascular complications in diabetic patients [3, 4]. Although these relationships have been well-established in adult populations, significant knowledge gaps remain concerning the interaction between insulin resistance and circulating progenitor cell populations, especially the dynamics of EPCs, in youth-onset T2DM. This unexplained link represents a key area for further exploration in understanding the rapid progression of cardiovascular disease seen in younger individuals with diabetes [15].

This study found that when participants were grouped into tertiles based on HOMA-IR levels, circulating stem cells and EPCs significantly decreased with increasing HOMA-IR tertiles, while other cardiovascular risk factors, such as BMI, SBP, DBP, TG, TC, and LDL-C, significantly increased, and HDL-C markedly decreased. No notable differences were found in terms of age, sex, FPG, or HbA1c levels. Correlation analysis revealed that circulating stem cells and EPCs were significantly positively correlated with HOMA-IR, BMI, SBP, DBP, TG, TC, and LDL-C, and negatively correlated with HDL-C, but showed no significant associations with age, sex, FPG, or HbA1c. After accounting for these cardiovascular risk factors, the partial correlation coefficient between HOMA-IR and circulating stem cells, as well as EPCs, was reduced but still maintained statistical significance. Regression analysis further revealed that when circulating stem cells and EPCs were used as dependent variables and HOMA-IR as the independent variable, the partial regression coefficient of HOMA-IR decreased after adjusting for BMI, SBP, DBP, TG, TC, LDL-C, and HDL-C. However, it still maintained statistical significance. These findings indicate that the decrease in circulating stem cells and endothelial progenitor cells (EPCs) observed in newly diagnosed adolescents with type 2 diabetes is linked to obesity, dyslipidemia, hypertension, and insulin resistance. Notably, HOMA-IR acts as an independent risk factor in this context. This result is consistent with earlier studies conducted on adults with type 2 diabetes [2-4]. A fundamental pathophysiological characteristic of type 2 diabetes is insulin resistance. Extended periods of insulin resistance before being diagnosed with type 2 diabetes can heighten the risk of cardiovascular disease. On the one hand, insulin resistance is strongly associated with conventional cardiovascular risk factors, including obesity, dyslipidemia, hyperglycemia, and hypertension, frequently suggesting the coexistence of these conditions. Conversely, insulin resistance specific to certain tissues (such as adipose tissue, liver, muscle, endothelial cells, and inflammatory cells) plays a role in the development of lipid disorders, increased blood glucose levels, chronic inflammation, and endothelial dysfunction. These factors, in turn, expedite the progression of atherosclerosis and vascular complications [9, 16]. This study revealed that reduced circulating stem cells and EPCs were not only significantly associated with HOMA-IR but also correlated with BMI, SBP, DBP, TG, TC, LDL-C, and HDL-C, while showing no significant relationship with age, sex, FPG, or HbA1c. Notably, age, sex, and hyperglycemia are established cardiovascular risk factors, yet no significant association with circulating stem cells and EPCs was observed here. This difference might arise because the study concentrated on children and adolescents who had recently been diagnosed with type 2 diabetes, meaning their exposure to these risk factors (such as age, sex, and glucose levels) was relatively brief. Moreover, the observed reduction in circulating stem cells and EPCs probably indicates markers of early-stage arterial pathology rather than signs of advanced vascular damage.

This study demonstrated that an increased HOMA-IR is independently associated with a decline in circulating stem cells and EPCs in adolescents newly diagnosed with T2DM. These findings identify insulin resistance as an independent risk factor for early vascular dysfunction in this demographic [17]. Although these findings underscore the clinical significance of monitoring cardiovascular risks associated with insulin resistance in youth-onset diabetes, the cross-sectional study design, small sample size, and sole reliance on circulating stem cells/EPCs as biomarkers of arterial pathology call for validation through large-scale prospective studies. Future studies should utilize thorough vascular evaluations and mechanistic analyses to elucidate the causal pathways connecting insulin resistance to early vascular remodeling in patients with youth-onset T2DM.

## CONCLUSION

In young individuals newly diagnosed with T2DM, insulin resistance—measured by HOMA-IR—is linked to lower levels of circulating stem cells and endothelial progenitor cells. This association remains significant even after accounting for factors like adiposity, blood pressure, and lipid profiles. This implies that insulin resistance might lead to early vascular dysfunction in this group by disrupting the mobilization of regenerative cells.

Our findings are subject to certain key limitations that constrain their interpretation. The cross-sectional nature of the design prevents drawing definitive conclusions regarding the causal relationship between insulin resistance and decreased levels of progenitor cells. Although HOMA-IR serves as a convenient surrogate measure, it might not comprehensively reflect insulin sensitivity at the tissue-specific level. Our study was based on cellular biomarkers; however, the absence of direct and comprehensive assessments of vascular function implies that the connection to clinical cardiovascular outcomes is still indirect. Unmeasured confounders, including patterns of physical activity, specifics of body composition, or particular inflammatory markers, might lead to residual bias. The fact that our study recruited Chinese youth from a single center may restrict the generalizability of our findings to other ethnic groups or geographic populations. Future longitudinal research is crucial for establishing causality and should include more accurate assessments of insulin sensitivity, comprehensive vascular phenotyping, and functional analyses of progenitor cells to elucidate the underlying mechanisms.

## Figures and Tables

**Table 1:** Clinical characteristics and levels of stem/progenitor cells across HOMA-IR tertile groups.

Characteristics	Group		χ^2^/F	P
	H1	H2 and H3		
No. of participants (M/F) Age (y) SBP (mmHg) DBP (mmHg) BMI (kg/m^2^) TG (mmol/L) TC (mmol/L) LDL-C (mmol/L) HDL-C (mmol/L) HbA1c (%) FPG (mmol/L HOMA-IR WBC10^3^ (/mL) CD34^+^/10^6^ (/mL) CD34^+^KDR^+^/10^6^ (/mL)	132(72/60) 18.9±4.1 108.7±10.3 70.9±6.6 23.4±3.2 1.25 ±0.23 4.23±0.49 2.55±0.45 1.39±0.41 7.9±1.8 8.01±0.81 2.2±0.30 5.1±0.4 369.5±30.3 21.1±2.8	128(72/56) 132(64/68) 19.3±3.9 19.7±4.4 117.4±11.4 128.1±13.1 78.4±6.9 81.9±8.0 24.9±2.9 25.9±3.4 1.37±0.30 1.49±0.29 4.52±0.64 4.75±0.71 2.77±0.53 2.96±0.64 1.20±0.19 1.12±0.21 8.2±2.0 8.1±1.9 8.16±0.79 8.18±0.82 3.7±0.33 4.9±0.34 4.9±0.3 5.3±0.2 315.4±28.4 269±26.8 16.5±2.1 11.9±1.9	- 0.456 3.672 16.421 3.489 5.192 6.232 5.231 4.174 0.191 0.249 12.818 0.212 7.362 4.763	- 0.709 0.000 0.000 0.032 0.006 0.003 0.007 0.016 0.814 0.771 0.000 0.687 0.000 0.000

**Abbreviations** HOMA-IR: homeostasis model assessment of insulin resistance; BMI: body mass index; SBP: systolic blood pressure; DBP: diastolic blood pressure; TG: triglyceride; TC: total cholesterol; LDL-C: low density lipoproteins cholesterol; HDL-C: high density lipoproteins cholesterol; FPG: fasting plasma glucose; HbA1c: hemoglobin A1c; WBC: White blood cells.

## Data Availability

The datasets generated and/or analyzed during the current study are available from the corresponding author upon reasonable request.

## LIST OF ABBREVIATIONS

BMI: Body Mass Index
CIMT: Carotid Intima-Media Thickness
CVD: Cardiovascular Disease
DBP: Diastolic Blood Pressure
EPCs: Endothelial Progenitor Cells
FINS: Fasting Insulin
FPG: Fasting Plasma Glucose
HbA1c: Glycated Hemoglobin
HDL-C: High-Density Lipoprotein Cholesterol
HOMA-IR: Homeostatic Model Assessment of Insulin Resistance
IR: Insulin Resistance
KDR: Kinase Insert Domain Receptor
LDL-C: Low-Density Lipoprotein Cholesterol
PBMCs: Peripheral Blood Mononuclear Cells
SBP: Systolic Blood Pressure
T2DM: Type 2 Diabetes Mellitus
TC: Total Cholesterol
TG: Triglycerides
WBC: White Blood Cells

## STANDARDS OF REPORTING

STROBE guidelines were followed.
